# Socially anxious individuals with low working memory capacity could not inhibit the goal-irrelevant information

**DOI:** 10.3389/fnhum.2013.00840

**Published:** 2013-12-06

**Authors:** Jun Moriya, Yoshinori Sugiura

**Affiliations:** ^1^Department of Psychology, College of Contemporary Psychology, Rikkyo UniversitySaitama, Japan; ^2^Graduate School of Integrated Arts and Sciences, Hiroshima UniversityHigashi-Hiroshima, Japan

**Keywords:** social anxiety, visual working memory capacity, goal setting, spatial blink, selective attention

## Abstract

Socially anxious individuals are interfered by distractors. Recent work has suggested that low working memory capacity and inappropriate temporary goal induce attention to distractors. We investigated the effects of working memory capacity and temporary goal on attention to distractors in social anxiety. Participants viewed a rapid serial visual presentation, in which participants reported the identity of a single target letter drawn in red. Distractors appeared before the target was presented. When the color of distractors was red (i.e., goal-relevant stimuli), low-capacity individuals were strongly interfered by the distractors compared to high-capacity individuals regardless of social anxiety. When the color of distractors was goal-irrelevant, low-capacity and high socially anxious individuals were strongly interfered by the distractors. These results suggest that socially anxious individuals with low working memory capacity could not inhibit the goal-irrelevant information and direct attention to distractors.

## INTRODUCTION

Anxious and socially anxious individuals tend to be more easily distracted by irrelevant stimuli. Although several previous studies have shown attentional prioritization of threatening distractors (e.g., threatening words, angry faces) among individuals with anxiety (Fox et al., 2005; Bar-Haim et al., 2007), recent research has shown that anxious and socially anxious individuals process non-emotional distractors as well (Derakshan et al., 2009; Moriya and Tanno, 2009a, 2010, 2011a; Ansari and Derakshan, 2010, 2011a,b; Sadeh and Bredemeier, 2011; Moser et al., 2012; Berggren and Derakshan, 2013). For example, Moriya and Tanno (2010) had participants search for a target letter (X or N) presented on an imaginary circle at a central fixation with a peripheral distractor. Although participants did not need to direct their attention toward the peripheral distractor, individuals high in social anxiety were more likely to attend to the distractor. Reaction times for the target were delayed for individuals high in social anxiety due to distractor interference. Few studies, however, have investigated why anxious and socially anxious individuals are distracted by irrelevant, non-emotional stimuli. In the present study, we focused on two important factors: visual working memory capacity (VWMC) and goal setting, both of which influence non-emotional distractor processing. We investigated the effects of these factors on distractor interference in individuals with social anxiety.

Previous studies have investigated individual differences in distractor processing, and suggest that individual differences in VWMC reflect spatial attention to distractors (Fukuda and Vogel, 2009, 2011). VWMC refers to the number of items an individual can represent in an on-line state; it is a limited ability (Luck and Vogel, 1997; Vogel and Machizawa, 2004; Awh et al., 2007). Several studies have suggested that individuals with low VWMC have poor attentional control and have difficulty filtering distractors (Vogel et al., 2005; McNab and Klingberg, 2008; Fukuda and Vogel, 2009, 2011). For instance, Fukuda and Vogel (2009, 2011) had participants perform a change detection task that measured VWMC while performing a visual task (e.g., spatial-blink task, visual search task) with a salient, to-be-ignored distractor. Individuals with low VWMC had difficulty filtering out distractors, and target detection performance subsequently suffered. Considering these previous results, we hypothesize that anxious and socially anxious individuals have low VWMC, which leads to distractor interference.

Interestingly, however, social anxiety is not necessarily associated with low VWMC. This association depends on the components of working memory. Social anxiety is negatively correlated with phonological working memory capacity (Amir and Bomyea, 2011; Visu-Petra et al., 2011), but positively correlated with VWMC (Moriya and Sugiura, 2012). According to Fukuda and Vogel (2009, 2011), enhanced distractor interference is associated with low VWMC. According to these results, socially anxious individuals with high VWMC should be able to efficiently filter out distractors. This, however, is inconsistent with previous research showing that anxious and socially anxious individuals do not ignore distractors (Derakshan et al., 2009; Moriya and Tanno, 2009a, 2010, 2011a; Ansari and Derakshan, 2010, 2011a,b; Sadeh and Bredemeier, 2011; Moser et al., 2012; Berggren and Derakshan, 2013). To address this issue, we need to assess the interactive effects of social anxiety and VWMC on distractor processing. Several previous studies have shown that cognitive control moderates the attentional prioritization of threatening distractors in anxiety, and an attentional bias toward threatening distractors has been observed among highly anxious individuals with low cognitive control (Derryberry and Reed, 2002; Peers and Lawrence, 2009; Reinholdt-Dunne et al., 2009; Susa et al., 2012). Thus, we predict that VWMC also moderates the interference of non-emotional distractors in social anxiety. In the present study, therefore, we investigated the interaction effect of VWMC and social anxiety on non-emotional distractor processing.

Distractor interference also depends on goal setting. When people set their goals for a specific feature (e.g., a red stimulus), a stimulus that has the same feature(s) as the goal(s) strongly attracts attention (Folk et al., 1992, 1994, 2002; Lamy et al., 2004; Anderson and Folk, 2012). Attentional priority is fully contingent on the top-down goal settings adopted by the observer, and goal-irrelevant distractors are simply suppressed. For example, in a study by Folk et al. (2002), participants viewed a central rapid serial visual presentation (RSVP), in which a target letter was defined as a particular color (e.g., red). Participants needed to detect a target letter while distractor letters were occasionally presented in the periphery prior to the presentation of the target. In this case, the red item comprised the attentional set. Attention to the peripheral distractors led to a decrement in target detection, in a phenomenon known as a *spatial blink*. When the color of the distractors differed from that of the target (e.g., target color was red and distractor color was blue), the effect of the spatial blink was still observed, but it was small. Although salient distractors attract attention (Theeuwes, 1992), goal-irrelevant distractors appear to have little ability to attract attention. On the other hand, when a distractor whose color matched the target’s color (e.g., both target and distractor color is red) was presented prior to the target’s appearance, the distractor captured attention, and the accuracy of target detection decreased; this decrement was much larger than in the case of goal-irrelevant distractors. Goal-relevant distractors strongly attracted attention compared to goal-irrelevant distractors. Moreover, attention to the goal-relevant distractors has been observed especially in individuals with low VWMC (Fukuda and Vogel, 2009). While attention to goal-irrelevant distractors derives from saliency, attention to goal-relevant distractors additionally depends on top-down control. It is, therefore, possible that anxious individuals’ goals have an effect on distractor processing.

A few previous studies have already shown the effects of goals on attentional priority in anxiety. Vogt et al. (2013) revealed an important role of goals on distractor processing in anxiety, even though the authors used emotional distractors. In this study, participants were asked to perform a dual task – a dot-probe task and a goal task – during each trial. During the goal task, participants were required to detect a specific picture (a goal-relevant picture) and respond as quickly as possible. During the dot-probe task, two pictures were presented simultaneously; immediately after the pictures disappeared, a probe appeared in one of two locations (either the same or opposite side of the preceding picture). Participants were asked to detect the location of the probe as quickly as possible. Notably, the goal-relevant picture was presented during the dot-probe task, even though it did not predict a probe location. When the goal-relevant picture and a threatening picture were presented simultaneously, highly anxious individuals did not direct attention toward the threatening stimulus but, rather, toward the goal-relevant picture. These results indicated that anxious individuals did not show distractor processing if their goal was to detect a specific target. Because Vogt et al. (2013) used emotional distractors, it is still unclear whether goals influence non-emotional distractor processing in social anxiety. Another open question is whether the effects of goals on distractor interference are influenced by VWMC.

In the present study, we investigated the effects of VWMC and goals on distractor processing in social anxiety. We focused on social anxiety because socially anxious individuals are hypervigilant to non-emotional visual information (Moriya and Tanno, 2009b), and cognitive control is strongly associated with trait social anxiety (Moriya and Tanno, 2008). In the present experiments, we used a spatial-blink task (Folk et al., 2002; Fukuda and Vogel, 2009). As mentioned above, we can measure the degree of attentional effects to distractors by target detection decrements, or *the spatial blink*. Previous studies have shown that the peripheral distractor produces a reduction in target identification accuracy when the distractor shares a target color (i.e., goal-relevant distractor) compared to when the color of the distractor differs from that of the target (Folk et al., 2002; Fukuda and Vogel, 2009). During this task, we can investigate the effects of goal setting on distractor processing. Fukuda and Vogel (2009) also showed that individuals with low VWMC had a large decrement in target detection during this task. The spatial-blink task is appropriate for investigating the interaction between VWMC and goals on distractor processing.

Our hypotheses were as follows. Basing our hypotheses on the results of Vogt et al. (2013), we assumed that participants would direct attention toward goal-relevant distractors regardless of whether they had social anxiety. Considering that individuals with low VWMC are hindered by goal-relevant distractors (Fukuda and Vogel, 2009), a decrement in target identification accuracy may be negatively correlated with VWMC, regardless of social anxiety (Hypothesis 1). However, for goal-irrelevant distractors, individuals high in social anxiety and low in VWMC may process distractors, since previous studies have shown that impaired cognitive control in anxiety increases attentional prioritization of goal-irrelevant distractors (Derryberry and Reed, 2002; Peers and Lawrence, 2009; Reinholdt-Dunne et al., 2009; Susa et al., 2012). Therefore, we hypothesized that when presented with goal-irrelevant distractors, a decrement in target identification may not be simply correlated with social anxiety, but may be associated with the interaction between social anxiety and VWMC (Hypothesis 2). Moreover, the decrement in target identification might be especially observed among individuals high in social anxiety but low in VWMC. The decrement in target identification may be bigger, along with the degree of social anxiety among individuals low in VWMC; however, this is unlikely to be the case among individuals high in VWMC (Hypothesis 3).

## MATERIALS AND METHODS

### PARTICIPANTS

Participants were 40 undergraduates (22 women) aged between 18 and 27 years (mean age = 19.5, SD = 2.0). Participants provided informed consent and had normal or corrected-to-normal vision.

### STIMULI AND PROCEDURE

#### Change detection task for visual working memory capacity

Participants first performed a change detection task (Luck and Vogel, 1997; Vogel and Machizawa, 2004)^1^. All stimulus arrays were presented within a 9.8° × 7.3° region on a monitor with a gray background, and stimuli were placed at least 2.0° (center to center) apart. Within the memory array, participants were presented with brief arrays of 4, 8, or 12 colored squares (0.65° × 0.65°) for 100 ms and asked to remember the items. Each square was selected at random from a set of seven highly discriminable colors (red, blue, violet, green, yellow, black, and white), and a given color could appear no more than twice within a single array. Memory was tested 1 s later by using a test array that was either identical to the memory array, or different by one color. Participants were required to press one of two buttons to indicate whether the two arrays were identical or different. The color of one item in the test array differed from the corresponding item in the memory array on 50% of the trials; the memory and test arrays were otherwise identical. Stimulus positions were randomized on each trial. There were 80 trials within each set size, providing participants with a total of 240 trials.

In order to investigate individual differences in memory capacity, we estimated each participant’s VWMC by *K*-estimates according to a standard formula (Cowan, 2001), *K* = *S* (*H* - *F*), where *K* is memory capacity, *S* is array size, *H* is observed hit rate, and *F* is false alarm rate. The hit rate is the proportion of correct responses when two arrays differ. The false alarm rate is the proportion of incorrect responses when two arrays are identical. *K* is computed in each set size. Considering that an average capacity of visual working memory is typically around three to four items (Luck and Vogel, 1997; Vogel and Machizawa, 2004), individual differences in VWMC might not be observed with low set sizes of less than four items. In order to capture individual differences, we focused on the average *K*-estimates for set sizes 8 and 12.

#### Spatial-blink task

**FIGURE 1:**
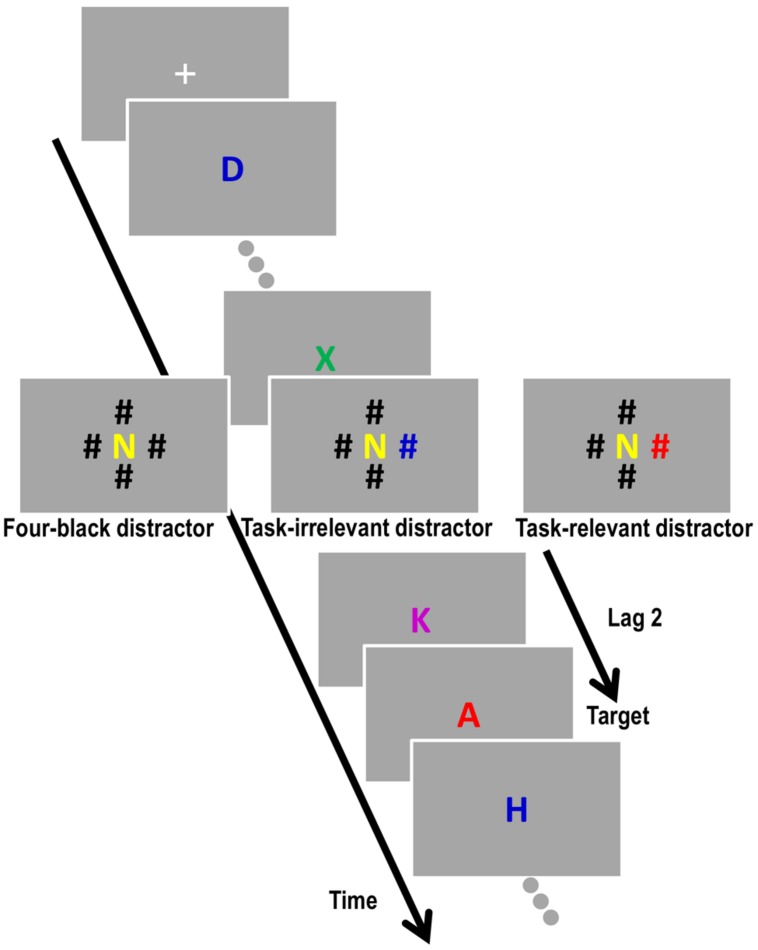
**Sequence of the spatial-blink task with a distractor-target lag of 2**.

After the change detection task, participants performed a spatial-blink task (Folk et al., 2002; Fukuda and Vogel, 2009). Participants observed a RSVP of colored letters (1.3° × 1.3°) presented at fixation (**Figure 1**). All the letters except for I, O, P, Q, and R were used to create a stream with 15 letters without repetition. One letter in the RSVP was red while the others were blue, green, yellow, or violet. Participants were required to identify a red letter – the target – in the RSVP. After a white fixation cross was presented in the middle of the screen for 500 ms, each letter was presented for 50 ms and followed by a 50-ms blank screen. For each RSVP, 15 letters were presented, and a target appeared equally often in positions 8 through 12 of the letter sequence.

There were four different distractor conditions. In the *no-distractor condition* (one-fourth of all trials), only central letters were presented, and each of the 15 frames in the RSVP contained only a central letter. In the distractor condition (three-quarters of all trials), four “#”s appeared 5.2° above, below, to the right, and to the left of a central letter, simultaneously with a target, or one, two, or three frames before the target letter. Depending on the distractor condition, the color of the “#”s differed. In the *four-black distractor condition* (one-third of the distractor trials), four black “#”s appeared. In the *goal-irrelevant distractor condition* (one-third of the distractor trials), three black “#”s and one colored “#” appeared. The colored “#” differed from the color of the target (blue, green, yellow, or violet). In the *goal-relevant distractor condition* (one-third of the distractor trials), three black “#”s and one red “#” appeared. That is, one of the “#”s was the same color as the target. The colored “#” was presented equally often in the four possible locations. Four-distractor conditions appeared randomly and equally often. Trials with four possible lags between the presentation of the target and the presentation of the distractors also appeared randomly and equally often. There were 80 trials within each distractor condition, providing participants with a total of 320 trials.

Both tasks were conducted on a 17-inch monitor. The experiments were programed using MATLAB equipped with the Psychophysics Toolbox (Brainard, 1997; Pelli, 1997). The viewing distance was about 60 cm.

#### Questionnaire

At the end of the task, participants completed the Japanese version of the Brief Fear of Negative Evaluation Scale (BFNE; Leary, 1983; Sasagawa et al., 2004). The BFNE assesses apprehension related to others’ negative evaluations and reflects one’s level of social anxiety. The scale consists of 12 items rated on 5-point Likert scales. The scale has high internal consistency (Cronbach’s alpha = 0.92) and high test–retest reliability with a 3-month interval (*r* = 0.74; Sasagawa et al., 2004).

## RESULTS

**FIGURE 2:**
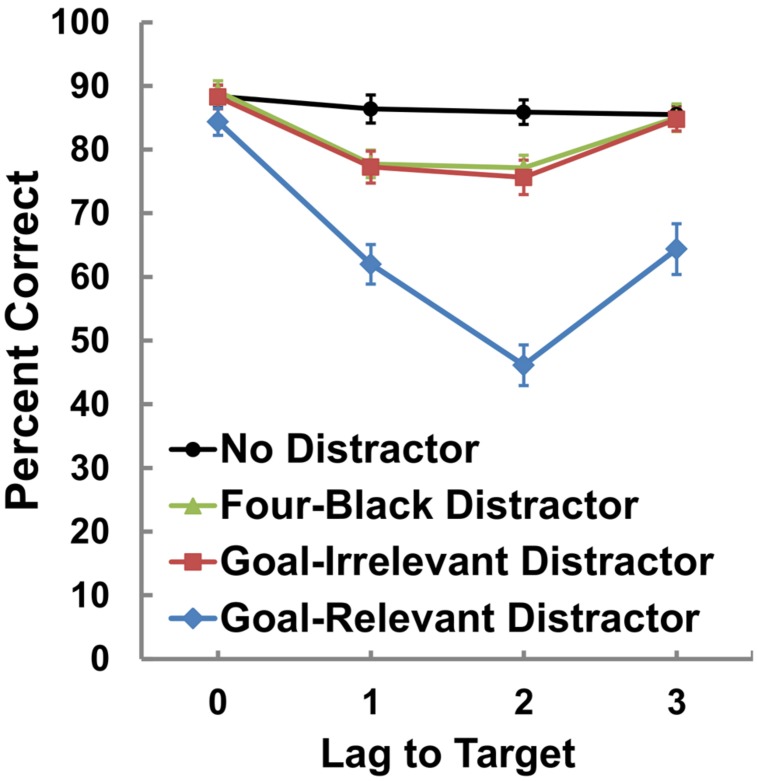
**Mean percentages of correct target identification**.

The mean percentages of correct target identifications within each distractor condition are presented in **Figure 2**. We used 4 (Distractor: no-distractor, four-black distractor, goal-irrelevant distractor, and goal-relevant distractor) × 4 (Lag: 0, 1, 2, and 3) ANOVAs to ascertain spatial blink. The analysis showed significant main effects of Distractor, *F*(3,117) = 87.57, *p* < 0.001, η^2^ = 0.69, and Lag, *F*(3,117) = 40.99, *p* < 0.001, η^2^ = 0.51. The two-way interaction was also significant, *F*(9,351) = 15.14, *p* < 0.001, η^2^ = 0.28. Further analyses revealed that under the four-black and goal-irrelevant distractor conditions, the mean percentages of correct identification were significantly lower at lags 1 and 2 than at lags 0 and 3 (*p* values < 0.01). Under the goal-relevant distractor conditions, the mean percentages of correct identification were also significantly lower at lags 1, 2, and 3 than at lag 0 (*p* values < 0.01). Moreover, the correct percentages at lag 2 were significantly lower than were those at lags 1 and 3 (*p* values < 0.01). The correct percentages under four-black and goal-irrelevant distractor conditions at lags 1 and 2 and goal-relevant distractor conditions at lags 1, 2, and 3 were significantly lower than were those under the no-distractor conditions (*p* values < 0.01). Specifically, correct target identification under the goal-relevant distractor condition at lag 2 was lower than during any other condition (*p* values < 0.01). These results suggest that spatial blink was observed in the present experiment, especially under the goal-relevant condition at lag 2.

We evaluated the decrement in target identification (i.e., the spatial-blink effect) by subtracting correct percentages under each distractor condition at lag 2 from the average correct percentages under the no-distractor condition. Next, we analyzed correlations between social anxiety, VWMC, and the effects of spatial blink on each distractor condition to investigate whether the decrement in target identification was negatively correlated with VWMC for goal-relevant distractors (Hypothesis 1), and not correlated with social anxiety for goal-irrelevant distractors (Hypothesis 2). Correlations are presented in **Table 1**. Social anxiety was positively correlated with VWMC. The spatial-blink effect under the four-black distractor condition was positively correlated with the spatial-blink effect under the goal-irrelevant and goal-relevant distractor conditions. Notably, the spatial-blink effect under the goal-relevant distractor condition was negatively correlated with VWMC. Individuals with high VWMC could inhibit spatial blink by goal-relevant distractors. However, social anxiety did not correlate with spatial blink under any other condition.

**Table 1:** Mean values, standard deviations, and correlations among social anxiety, visual working memory capacity, and decreased percentages of target identification.

	VWMC	Four-black	Goal-irrelevant	Goal-relevant	Average	SD
Social anxiety	0.35^*^	0.23	0.25	-0.08	40.7	8.5
VWMC	-	-0.02	-0.09	-0.33^*^	3.9	1.1
Four-black		-	0.50^**^	0.33^*^	10.5	12.3
Goal-irrelevant			-	0.17	10.9	13.1
Goal-relevant				-	40.4	19.8


* *p* < 0.05, **p < 0.01.

VWMC, visual working memory capacity; four-black, four-black distractor condition; goal-irrelevant, goal-irrelevant distractor condition; goal-relevant, goal-relevant distractor condition.

We also analyzed partial correlations between social anxiety, VWMC, and the effects of spatial blink when controlling for social anxiety and VWMC, respectively (**Table 2**). When controlling for social anxiety, VWMC was marginally correlated with the spatial-blink effect under the goal-relevant distractor condition, but was not clearly significant, *r* = -0.31, *p* = 0.059. When controlling for VWMC, social anxiety was not significantly correlated with the spatial-blink effect under the goal-irrelevant distractor condition, although it was marginally significant, *r* = 0.29, *p* = 0.074.

**Table 2:** Partial correlations controlling for social anxiety and visual working memory capacity.

	Social anxiety	Four-black	Goal-irrelevant	Goal-relevant
VWMC	–	-0.25	-0.25	-0.31
Four-black	0.15	–	0.44^**^	0.33^*^
Goal-irrelevant	0.29	0.43^**^	–	0.20
Goal-relevant	0.01	0.27	0.13	–

* *p* < 0.05, **p < 0.01.

*Partial correlations controlling for social anxiety are above the diagonal and partial correlations controlling for visual working memory capacity are below the diagonal.*

**FIGURE 3:**
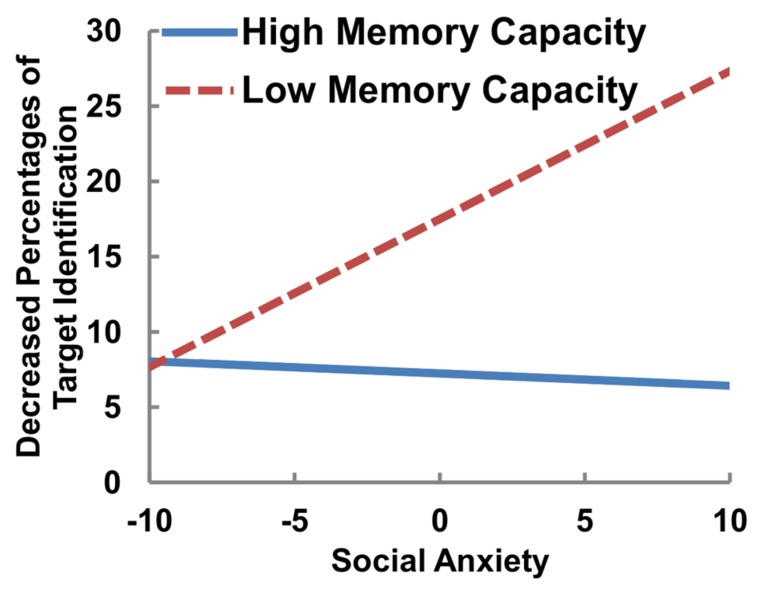
**Conditional associations between social anxiety and decreased percentages of target identification for high and low visual working memory capacity**.

To investigate the interaction effects between social anxiety and VWMC on the decrement in target identification for goal-irrelevant distractors (Hypothesis 2), we focused on the moderating role of working memory capacity on the link between social anxiety and spatial blink. We applied general linear models predicting spatial-blink effects by social anxiety and working memory capacity. First, all independent variables were centered on the grand mean, because mean centering has interpretational and computational advantages (Aiken and West, 1991; Bauer and Curran, 2005). In Step 1 (main effects), social anxiety scores and VWMC were entered, and then in Step 2 (interaction effect), the social anxiety × memory capacity interaction was entered for each distractor condition. The results of the regression analysis are shown in **Table 3**. Under the four-black and goal-relevant distractor conditions, there were no significant main effects or any interaction effects. Under the goal-irrelevant distractor condition, the main effect was not significant. However, the interaction was significant, as was the model, *F*(3,36) = 3.46, *R*^2^ = 0.22, *p* < 0.05. The interaction is depicted in **Figure 3** using a simple slope analysis at one SD above and below the mean VWMC (Preacher et al., 2006) in order to examine whether the decrement in target identification increases along with the degree of social anxiety among individuals low in VWMC (Hypothesis 3). The simple slope for high VWMC was significant (*B* = 0.98, β = 0.61, *t* = 2.99, *p* < 0.01) whereas that for low VWMC was not (*B* = -0.08, β = -0.05, *t* = -0.23, *p* > 0.80).

**Table 3:** Summary of the hierarchical regression analysis for social anxiety and visual working memory capacity predicting the effects of spatial blink on each distractor condition.

	Four-black				Goal-irrelevant				Goal-relevant			
	*B*	SE *B*	β	Δ*R*^2^	*B*	SE *B*	β	Δ*R*^2^	*B*	SE *B*	β	Δ*R*^2^
Step 1				0.07				0.11				0.10
SA	0.19	0.21	0.16		0.49	0.27	0.31		0.02	0.40	0.01	
VWMC	-2.58	1.61	-0.27		-3.28	2.07	-0.26		-6.11	3.13	-0.32	
Step 2				0.04				0.11^*^				0.02
SA	0.18	0.20	0.14		0.45	0.25	0.28		0.04	0.41	0.02	
VWMC	-3.34	1.69	-0.35		-4.91	2.08	-0.39^*^		-5.23	3.35	-0.28	
SA × VWMC	-0.24	0.18	-0.23		-0.51	0.22	-0.37^*^		0.28	0.35	0.13	
Total *R*^2^				0.11				0.22^*^				0.12

* p < 0.05, SA, social anxiety.

## DISCUSSION

In the present experiment, we investigated the effects of VWMC and goals on distractor processing in individuals with social anxiety. Our results showed that regardless of the degree of social anxiety, individuals with low VWMC had difficulty in inhibiting the processing of goal-relevant distractors. For goal-irrelevant distractors, however, we found an interaction between VWMC and social anxiety. Individuals with high social anxiety and low VWMC showed strong interference from goal-irrelevant distractors, whereas individuals with high social anxiety and high VWMC, and individuals with low social anxiety, did not show strong interference. Even under non-color distractor trials (i.e., four-black distractor trials), participants showed a decrement in target identification accuracy compared to the no-distractor trials, although this decrement was not associated with social anxiety and VWMC. When presented with goal-irrelevant but salient colored distractors under goal-irrelevant conditions, socially anxious individuals with low VWMC had difficulty filtering out distractors.

Under goal-relevant distractor conditions, attention to distractors was associated with low VWMC, regardless of the degree of social anxiety. This result is consistent with previous research (Fukuda and Vogel, 2009, 2011) and our hypothesis (Hypothesis 1). Individuals with low VWMC could not filter out the goal-relevant distractors efficiently. Because attention may be allocated to the location of peripheral distractors for some time, these individuals miss the central target, which is presented soon after the onset of the distractors. However, the association between VWMC and attention to goal-relevant distractors was not clearly observed when controlling for the degree of social anxiety. Considering that the main effect of multiple regression analysis under the goal-relevant condition was not significant (either when entering social anxiety, or VWMC), attention to goal-relevant distractors was influenced by social anxiety. However, because the partial correlation between social anxiety and the decrement in target identification under the goal-relevant condition did not reach significance, and the regression coefficient of social anxiety was too small, social anxiety alone may have little effect on attention to goal-relevant distractors.

Social anxiety itself was not correlated with goal-irrelevant distractor processing; socially anxious individuals with low VWMC, however, did show goal-irrelevant distractor processing. This is consistent with our hypotheses (Hypotheses 2 and 3). Previous studies have shown that socially anxious individuals experience interference from distractor stimuli, whereas impaired cognitive control among individuals with anxiety moderates interference from threatening distractors (Derryberry and Reed, 2002; Peers and Lawrence, 2009; Reinholdt-Dunne et al., 2009; Susa et al., 2012). In some studies, however, VWMC – being an aspect of cognitive control – was not diminished but enhanced among individuals with social anxiety (Moriya and Sugiura, 2012). Therefore, we were interested in assessing the interactive effects of social anxiety and VWMC on distractor interference. The present results showed that even highly socially anxious individuals with high VWMC could efficiently filter out goal-irrelevant distractors, but individuals with high social anxiety and low VWMC could not.

Social anxiety was not associated with distractor interference for goal-relevant distractors, but was associated with interference from goal-irrelevant distractors. According to Vogt et al. (2013), even anxious individuals direct attention toward goal-relevant stimuli. Our findings are consistent with their results. One difference in the present study was that socially anxious individuals with low VWMC could not suppress goal-irrelevant distractors, whereas in Vogt et al. (2013), highly anxious individuals did not attend to goal-irrelevant stimuli. This may have occurred due to differences in the study tasks. In Vogt et al.’s (2013) study, a goal-irrelevant stimulus was presented alongside a goal-relevant stimulus. While their results suggest that anxious individuals can direct attention to goal-relevant targets, such findings do not mean that their participants could suppress goal-irrelevant distractors. In the present study, we showed goal-relevant and goal-irrelevant distractors during each trial. The present results suggest that individuals high in social anxiety have difficulty filtering out goal-irrelevant distractors. However, socially anxious individuals may be able to reduce the effects of goal-irrelevant distractors if they have high VWMC.

However, in the four-black distractor trials, there was no interactive effect between social anxiety and VWMC on distractor interference. The four-black distractors were also goal-irrelevant distractors. Four letters were black during these trials, while one letter was colored in the goal-irrelevant distractor conditions. The colored letter was more salient compared to other three black letters. The salient distractor may attract attention for individuals with social anxiety. Previous studies have shown that anxious and socially anxious individuals are sensitive to non-emotional salient stimuli, and exogenously direct attention toward these distractors (Moriya and Tanno, 2009a; Moser et al., 2012). The present results suggest that socially anxious individuals with low VWMC experience interference from particularly salient distractors. Under the four-distractor conditions, only the central letter was colored, and distractors were not salient. Therefore, we did not find any effects of social anxiety on distractor interference under the four-distractor conditions.

The present results have valuable clinical implications. One of the key issues in social anxiety disorder is the processing of goal-irrelevant emotional distractors (Mogg et al., 2004). Because attentional maintenance to goal-irrelevant threatening stimuli increases anxiety, many clinical researchers are optimistic about the potential use of attentional bias modification, in which individuals with social anxiety disorder are trained to disengage attention from goal-irrelevant threatening stimuli (Schmidt et al., 2009). However, it is difficult to disengage or avert attention from goal-irrelevant threatening stimuli. The present results showed the possibility that increasing VWMC is a useful training method for efficient disengagement from goal-irrelevant threatening stimuli. In the present results, even highly socially anxious individuals could ignore the goal-irrelevant stimuli if they were also had high VWMC. Although further research must be undertaken to reveal whether the present results are observed for goal-irrelevant *emotional* distractors, it would also be valuable to investigate whether increasing VWMC in clinical samples could enhance suppression of goal-irrelevant distractors and decrease their anxiety.

Although this is the first study to show the interactive effects of VWMC and social anxiety on non-emotional distractor processing, some limitations should be noted. First, the present study demonstrated attentional processing of goal-irrelevant distractors in social anxiety with low VWMC, but we could not divide the effects of attentional capture to – vs. attentional disengagement from – goal-irrelevant distractors. Two possibilities are responsible for the present results. The first is that individuals with high social anxiety and high VWMC can resist attentional capture to goal-irrelevant distractors. The second is that individuals with high social anxiety and high VWMC also direct attention to goal-irrelevant distractors, but can efficiently disengage from these distractors. It is very important to investigate the effects of these two attentional systems, since such systems have different effects on generalized and social anxiety. Recent studies have shown impaired attentional disengagement in general anxiety and social anxiety (Fox et al., 2001, 2002; Yiend and Mathews, 2001; Amir et al., 2003; Georgiou et al., 2005; Koster et al., 2006; Moriya and Tanno, 2011b; Wieser et al., 2012). Considering our results together with such instances of previous research, we posit that individuals with high social anxiety and low VWMC do not disengage from goal-irrelevant distractors while showing a decrement in target identification. This interpretation is consistent with Fukuda and Vogel (2011), who showed that low VWMC was not related to attentional capture to distractors but, rather, impaired attentional disengagement from distractors. Future studies should assess these two different effects of attentional capture and disengagement on distractor processing in anxiety. Second, we did not investigate the effects of other scales (e.g., trait and state anxiety, depression) in the present study, and used a single measure of social anxiety. It is still unclear whether distractor processing is also influenced by the interaction between VWMC and, for example, trait anxiety. Many scales of negative emotionality should be used in future studies. Third, our sample size was not sufficient to investigate individual differences. Further studies should include a larger number of participants in order to corroborate the present results.

In summary, the present study investigated the effects of goal setting and VWMC on distractor processing during a spatial-blink task among socially anxious participants. Participants processed the goal-relevant distractors regardless of social anxiety, and displayed a spatial blink. For goal-irrelevant distractors, distractor processing was also observed, but this was associated with an interaction between VWMC and social anxiety. Individuals with high social anxiety but low VWMC exhibited strong distractor interference; meanwhile, those with high social anxiety and high VWMC, as well as those with low social anxiety, did not show strong interference. Although it is still unclear whether the present results are specific to social anxiety or any other negative emotionality (e.g., trait and state anxiety, depression), the present results indicate that it is important to consider the effects of goals and VWMC on distractor processing in social anxiety.

## Conflict of Interest Statement

The authors declare that the research was conducted in the absence of any commercial or financial relationships that could be construed as a potential conflict of interest.

## Footnotes

1 Data related to visual working memory capacity in the present study is part of data obtained from a previous study (Moriya and Sugiura, 2012). However, in that study, we did not analyze the data with the results of a spatial-blink task.
